# Dr. Albert Merritt Cook

**Published:** 1914-11

**Authors:** 


Dr. Albert Merritt Cook, Buffalo, 1881, Bellevue, 1883, for 23 years a practitioner of Newcastle, Pa., died at his home in Pasadena, Calif., August 16, aged 59.

